# Case report: A Chinese patient with glutathione synthetase deficiency and a novel glutathione synthase mutation

**DOI:** 10.3389/fped.2023.1212405

**Published:** 2023-07-27

**Authors:** Xiaojiao Wu, Jiancheng Jiao, Yaofang Xia, Xiaotong Yan, Zehao Liu, Yanyan Cao, Li Ma

**Affiliations:** ^1^ Department of Neonatology, Children’s Hospital of Hebei Province, Shijiazhuang, China; ^2^ Pediatric Clinical Research Centre of Hebei Province, Children's Hospital of Hebei Province, Shijiazhuang, China; ^3^ Institute of Pediatric Research, Children's Hospital of Hebei Province, Shijiazhuang, China

**Keywords:** glutathione synthetase deficiency, 5-oxoprolinuria, glutathione synthetase gene variation, newborn, inherited metabolic disease, case report

## Abstract

Glutathione synthetase deficiency (GSSD) is an autosomal-recessive metabolic disorder caused by glutathione synthetase (*GSS*) gene mutations. No more than 90 cases of GSSD have been reported worldwide; thus, the spectrum of *GSS* mutations and the genotype–phenotype association remain unclear. Here, we present a severely affected infant carrying a compound heterozygous *GSS* variation, c.491G > A, and a novel variant of c.1343_1348delTACTTC. We also summarize the clinical manifestations, treatment protocol, prognosis, and genetic characteristics of previously reported GSSD cases in China. In this case study, our patient presented with tachypnea, jaundice, intractable metabolic acidosis, and hemolytic anemia. Urinary-organic acid analysis revealed elevated 5-oxoproline levels. Further, this patient showed improved outcomes owing to early diagnosis and the timely administration of vitamins C and E. Therefore, our study indicates that in clinical cases of unexplained hemolytic anemia and metabolic acidosis, GSSD should be considered. Additionally, genetic testing and antioxidant application might help identify GSSD and improve the prognosis.

## 1. Introduction

**Figure 1:**
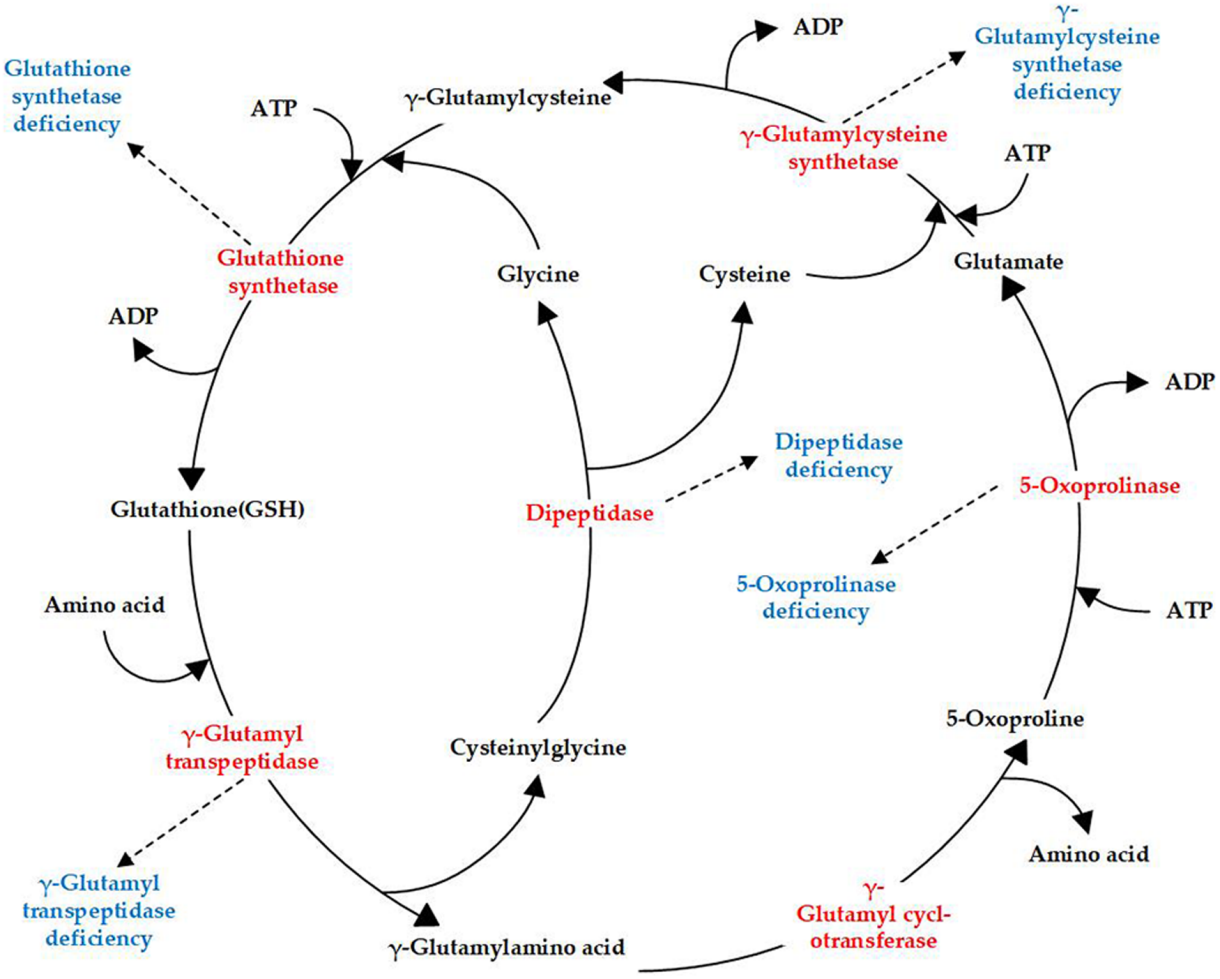
γ-Glutamyl cycle and enzyme deficiencies (highlighted in red) related to different diseases (highlighted in blue).

Glutathione synthetase deficiency (GSSD; OMIM 266130) is a rare autosomal-recessive metabolic disorder characterized by decreased glutathione (GSH) levels owing to defects in glutathione synthetase (GSS). GSH, a tripeptide consisting of glutamate, cysteine, and glycine, is common in mammalian cells. GSH is synthesized in a two-step reaction catalyzed by γ-glutamylcysteine synthetase and GSS in the γ-glutamyl cycle, which includes an additional four enzymes (Figure 1). Currently, genetic deficiencies have been reported for five of the enzymes in the cycle: γ-glutamylcysteine synthetase, GSS, γ-glutamyl transpeptidase, 5-oxoprolinase, and dipeptidase (1). These deficiencies lead to distinct metabolic disorders, with GSSD being the most frequently encountered GSH metabolic disorder (Figure 1).

Patients with GSSD, which is classified as mild, moderate, or severe based on clinical symptoms, usually show disease onset symptoms during the neonatal period (2). Specifically, patients with mild GSSD only show compensatory hemolytic anemia, whereas those with moderate or severe GSSD develop hemolytic anemia, metabolic acidosis, and persistent 5-oxoprolinuria. In addition, patients with severe GSSD develop progressive nervous system dysfunctions, such as intellectual disability, seizures, and ataxia, and may also show recurrent bacterial infections (2), pathological electroretinograms, and retinal pigmentation (3). To date, no more than 90 GSSD cases have been reported worldwide (4, 5), and approximately 25% of these patients die during the neonatal period owing to infection or electrolyte disturbance (6). Here, we present a case study of a neonate with severe GSSD carrying c.491G > A (p.?) and c.1343_1348delTACTTC (p.L448_L449del) variants, and summarize the previously reported GSSD cases in China. Our objective in this study was to expand the varietal spectrum and explore genotype–phenotype correlations. We expect that such findings can serve as a basis for accurate prognosis and genetic counseling.

## 2. Case description

A 6-day-old girl, presenting with feeding difficulties for 6 days, as well as tachypnea and metabolic acidosis for 2 days, was referred to our hospital. She was born at 38+5 weeks of gestation by cesarean section due to abnormal umbilical artery flow from a gravida 2, parity 2 (G2P2) mother. Her birth weight was 2,400 g, while her Apgar score was unknown. The mother's blood type was determined to be O and hers was B. Further, her brother and parents were found to be healthy and her parents were non-consanguine. This patient had no dysmorphic features. On day 2 of life, she was admitted to the neonatal department of a birth hospital with jaundice. Analysis of her liver function showed a total bilirubin level of 101.1 µmol/L (0–17.0 µmol/L), direct bilirubin level of 10.3 µmol/L (0–8.0 µmol/L), reticulocyte count of 10.42% (2.0%–6.0%), and she was negative in the Coombs test. Phototherapeutics was administered to reduce the yellowing of her skin, and immunoglobulin was used to inhibit hemolysis. The patient was nourished intravenously. On day 3 after birth, her cranial ultrasound revealed bilateral subependymal cystic cavities, and on day 4, she developed tachypnea. Blood-gas analysis showed metabolic acidosis and a blood-ammonia level of 85.4 µmol/L (18–72 µmol/L). Additionally, routine blood testing showed a hemoglobin level of 77 g/L (110–160 g/L). Thus, the patient was referred to our department for further treatment, given that the treatment of acidosis and anemia at the birth hospital resulted in no improvements.

On admission, her vital signs were as follows: temperature, 36.2°C; heart rate, 100/min; respiratory rate, 70/min; and blood pressure, 111/62 mm Hg. She exhibited low autonomic activity, diminished primitive reflexes, yellowish skin, muscular hypotonia, and poor peripheral blood circulation. Further, blood-gas analysis on admission also revealed metabolic acidosis (pH, 7.340; PCO_2_, 19.9 mmHg; PO_2_, 70.3 mmHg, HCO_3_^−^, 10.5 mmol/L; Base Excess-, 13.4 mmol/L; and O_2_ saturation, 95.6%). The infant still had symptoms of anemia (hemoglobin level, 103.6 g/L [110–160 g/L], reduced red blood cell-specific volume, 33.1% [49%–60%], and showed increased total serum bilirubin levels [104 µmol/L (0–17.0 µmol/L)]. However, her lactic acid level was normal.

**Figure 2:**
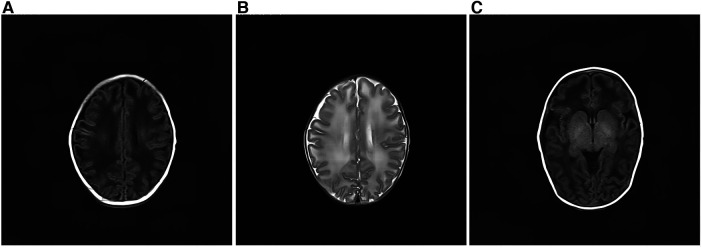
Cranial magnetic resonance imaging of the patient. (**A**) There was a wide range of long T1 signals in the bilateral frontotemporal parietal lobes. The lesions on both sides are roughly symmetrical. (**B**) The bilateral frontotemporal parietal lobes had a wide range of long T2 signals. The lesions on both sides are roughly symmetrical. (**C**) Short T1 signals can be seen in the bilateral pallidum.

After admission, the infant had irregular breathing and was administered humidified high flow nasal cannula (HHNFC), which was discontinued when her breathing improved. Further, she had hemolytic anemia and was transfused with suspended red blood cells to correct this condition. She also had a high total serum bilirubin level and showed yellow skin staining, indicative of jaundice; therefore, intermittent phototherapy was administered as treatment in this regard until the skin staining subsided. The infant's blood-gas levels were monitored several times, and the findings suggested the presence of intractable metabolic acidosis. Thus, sodium hydrogen carbonate was administered intravenously to correct the acid–base imbalance, after which the treatment was gradually changed to oral administration. On day 11 of admission, the analysis of her blood amino acid levels and her acylcarnitine spectrum showed no significant abnormalities. However, urine-organic acid analysis showed a high 5-oxoproline level [1,100 (normal range: 0–10.0)], suggestive of a defect in GSH metabolism. On day 13 after admission, amplitude-integrated electroencephalography did not show any significant abnormalities. However, cranial magnetic resonance imaging showed a wide range of long T1 and T2 signals in the bilateral frontotemporal parietal lobes, short T1 signals in the bilateral pallidum (Figure 2).

**Figure 3:**
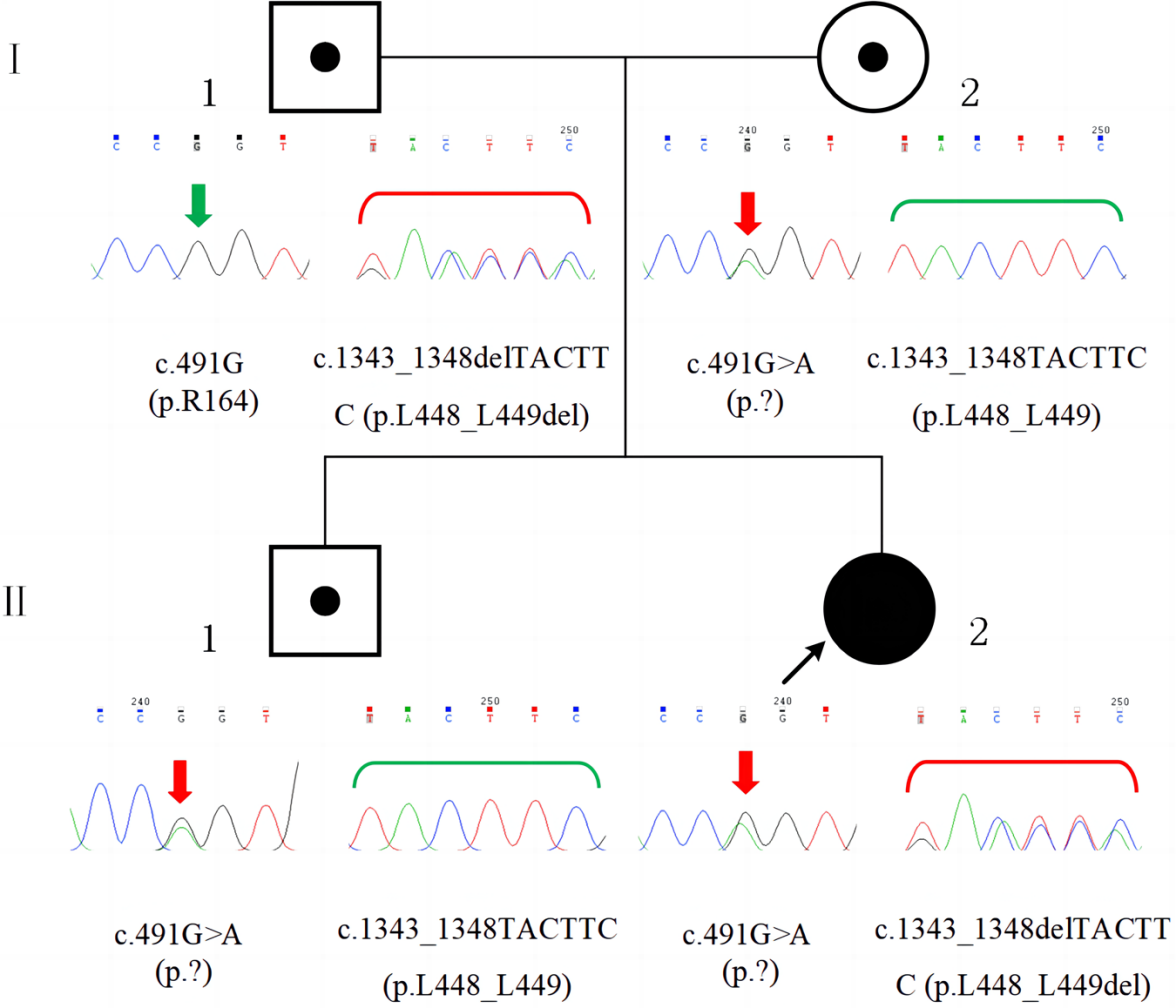
Family pedigree and *GSS* variants confirmed by sanger sequencing. The patient (II-2) had compound heterozygous variants, c.491G > A (p.?) and c.1343_1348delTACTTC (p.L448_L449del), which were inherited from her mother (I-2) and father (I-1), respectively. Her brother (II-1) had the c.491G > A (p.?) variant, which was inherited from his mother (I-2).

Compound heterozygous variants in *GSS* were detected in the proband by performing whole-exome sequencing (Beijing Fulgent Technologies Inc., Beijing, China): A c.491G > A (p.?) variant was found in exon 5, and a c.1343_1348delTACTTC (p.L448_L449del) variant was found in exon 13. Sanger sequencing confirmed that the c.491G > A (p.?) and c.1343_1348delTACTTC (p.L448_L449del) variants were inherited from the mother and father, respectively (Figure 3). Her brother carried only the c.491G > A (p.?) variant. The c.491G > A (p.?) variant was classified as pathogenic (PM2_Supporting + PM3_VerySrong + PS3+ PP4), and the c.1343_1348delTACTTC (p.L448_L449del) variant was classified as likely pathogenic (PM2_Supporting + PM3 + PM4 + PP4), according to the American College of Medical Genetics and Genomics guidelines and Sequence Variant Interpretation Recommendations by ClinGen. A diagnosis of GSSD was finally made based on intractable metabolic acidosis, jaundice, hemolytic anemia, hyperbilirubinemia, massive 5-oxoprolinuria, and the presence of compound heterozygous variants in *GSS*.

After the diagnosis of GSSD, the infant continued to receive l-carnitine to improve her metabolism, oral sodium bicarbonate to correct acidosis, and oral vitamins C and E to prevent oxidative stress. On day 19 of admission, the infant, now generally in good condition with the metabolic acidosis corrected, was discharged. Thereafter, the parents were instructed to regularly review the blood-gas analysis and cranial ultrasound data, adjust the doses of various oral medications, and monitor the infant's growth and development. At the first follow-up, the infant was nearly 6 months old, with an average weight and height, and was in good general condition. Her urine-organic acid analysis indicated an increase in 5-oxoproline levels, although the difference was not statistically significant compared with that of previously observed values. At the time of this writing, the child was 2 years old, weighed 11.3 kg [1 standard deviation (SD) below the mean], and was 81.2 cm tall (between 2 and 1 SD below the mean). Additionally, she had a head circumference of 46 cm (1 SD below the mean), mild anemia (hemoglobin: 104 g/L), mild compensatory metabolic acidosis (pH, 7.431; Base Excess-: 6 mmol/L), and her urine-organic acid analysis showed an increase in 5-oxoproline level [400.4 (normal range: 0–10.0)]. Further, she had no history of recurrent infection, and fundus screening revealed retinal pigmentation. Her language and gross motor development were moderately delayed. She was treated daily with oral sodium bicarbonate, vitamin C, and vitamin E.

## 3. Discussion

**Figure 4:**
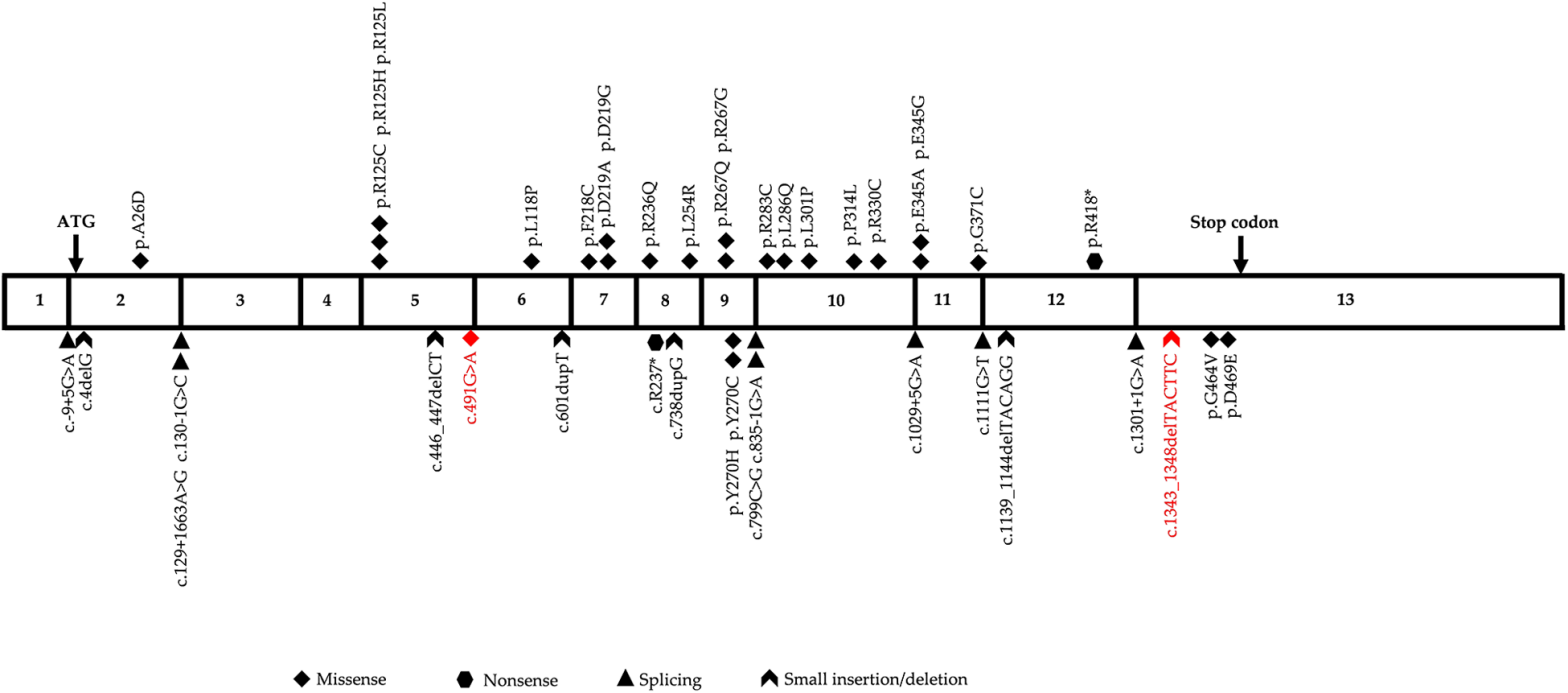
Diagrammatic representation of human glutathione synthetase (*GSS*) cDNA sequence. The two variants identified in this study are highlighted in red.

GSSD is caused by genetic variations in *GSS*, which is located on chromosome 20q11.2, contains 13 exons, and is approximately 32 kb in length. The Human Gene Mutation Database (HGMD) contains 43 different *GSS* variants that have been reported as of January 2023, with missense variants showing predominance (55.8%), followed by splice variants (21.0%), small insertions/deletions (11.5%), large insertions/deletions (7.0%), and nonsense variants (4.7%). The variant sites are distributed throughout the complementary DNA with no obvious concentrated distribution sites (Figure 4). To date, eight different variants, including three frameshift mutations (37.5%), two missense mutations (25.0%), one nonsense mutation (12.5%), one copy number mutation (12.5%), and one six-base in-frame deletion (12.5%), have been reported in China in 10 patients from nine unrelated families (Table 1). In this study, we identified two heterozygous variants, c.491G > A (p.?) and c.1343_1348delTACTTC (p.L448_L449del), in a newborn with severe GSSD. The former variant, which affects gene splicing (11–13), was inherited from the mother, whereas the latter variant, which has not been previously reported, was inherited from the father.

**Table 1:** GSS variations in Chinese patients with glutathione synthetase deficiency (GSSD).

Patient number (Ref.)	Mutation at the nucleotide level	Mutation at the protein level	Exon	Mutation type	Frequency	Phenotype
1 (7)	c.491G > A	p.?	5	Heterozygous	60% (12/20)	Severe
	c.446_447del	p.S149fs	5		5% (1/20)	
2 (8)	c.491G > A	p.?	5	Heterozygous	60% (12/20)	Moderate
	c.847C > T	p.R283C	10		5% (1/20)	
3 (8)	c.491G > A	p.?	5	Homozygous	60% (12/20)	Moderate
4 (9)	c.491G > A	p.?	5	Homozygous	60% (12/20)	Moderate
5 (10)	c.491G > A	p.?	5	Heterozygous	60% (12/20)	Severe
	c.1252C > T	p.R418X	12		5% (1/20)	
6 (10)	c.491G > A	p.?	5	Homozygous	60% (12/20)	Moderate
7 (10)	c.491G > A N/A	p.? N/A	5 N/A	Heterozygous	60% (12/20) N/A	Severe
8 (5)	c.738dupG9	p.S247fs	8	Heterozygous	5% (1/20)	Severe
	chr20:33530733–33530809	N/A	3		5% (1/20)	
9 (11)	c.491G > A	p.?	5	Heterozygous	60% (12/20)	Severe
	c.800G > A	p.R267Q	9		5% (1/20)	
10 [This study]	c.491G > A	p.?	5	Heterozygous	60% (12/20)	Severe
	c.1343_1348delTACTTC	p.L448_L449del	13		5% (1/20)	

N/A, data not available.

Variants in *GSS* cause GSS protein deficiency or reduced GSS activity, resulting in lower GSH levels. As an antioxidant, GSH is involved in several important biological functions, such as scavenging free radicals in the body, conferring resistance to oxidative stress, and maintaining cell membrane integrity. GSH is normally present at high levels in red blood cells; thus, decreased levels can make them more susceptible to damage from oxidative stress, leading to rupture and causing hyperbilirubinemia and anemia (6). GSH can also regulate its own biosynthesis by inhibiting γ-glutamylcysteine synthetase. Notably, when the GSH level decreases, impaired negative-feedback inhibition leads to the accumulation of γ-glutamylcysteine, which is converted to 5-oxoproline via the γ-glutamylcyclo-transferase pathway. The 5-oxoproline accumulates in body fluids or is excreted via urine (14), inducing metabolic acidosis and 5-oxoprolinuria. GSH can also function as a neuromodulator or neurotransmitter (15), reducing GSH synthesis may cause neurological pathologies. Moreover, the increased susceptibility to bacterial infections in patients with GSSD is due to defective granulocyte function (1).

GSSD can be diagnosed based on clinical presentation, an elevated urinary 5-oxoprolinuria concentration, and low GSS activity in erythrocytes or fibroblasts. Our patient was diagnosed with severe GSSD after neonatal onset, with symptoms including hemolytic anemia, hyperbilirubinemia and severe metabolic acidosis (intractable), urine-organic acid analysis results suggestive of the presence of 5-oxoprolinuria, and imaging results suggestive of central nervous system damage and developmental retardation in the infant at follow-up. Six severe and four moderate cases of GSSD have been reported in China; no mild cases have been reported (Table 1). Three of the six patients with severe disease died, while two showed severe neurodevelopmental impairment, and one case was not followed-up. Further, three of the four patients with moderate disease had good prognoses and showed normal neurodevelopment; no data were available on the one remaining case with moderate GSSD (Table 2). However, the occurrence of 5-oxoprolinuria is associated with various factors, such as dietary habits, drug metabolism, and inherited metabolic disorders. Hereditary 5-oxoprolinuria may also be due to 5-oxoprolinase deficiency (1) owing to mutations in *OPLAH*. However, in this case, the patients usually do not show symptoms of metabolic acidosis or hemolytic anemia, and antioxidant therapies are ineffective (10). Genetic analysis enables the rapid and accurate diagnosis of the cause of hereditary 5-oxoprolinuria.

**Table 2:** Clinical features and laboratory data corresponding to patients diagnosed with GSSD in China.

Patient number (Ref.)	Sex	Age at onset	Clinical presentation	Hemolytic anemia	Metabolic acidosis	Urinary 5-oxoproline	Cranial MRI/CT	Treatment	Outcome
1 (7)	F	1 day	Tachypnea, feeding deficiency, dyspnea, and jaundice	Yes	Yes	513.420 (0–7.6) mmol/mol Cr	N/A	L-carnitine; calcium leucovorin; sodium bicarbonate; and vitamins B1, B12, C, E, A, and D	Died after 12 days
2 (8)	N/A	After birth	Tachypnea and poor spirit	Yes	Yes	316.4 (0–7.6) µmol/L	N/A	N/A	Normal psychomotor development
3 (8)	N/A	After birth	Tachypnea and poor spirit	Yes	Yes	210.5 (0–7.6) µmol/L	N/A	N/A	Normal psychomotor development
4 (9)	M	After birth	Tachypnea and poor spirit	N/A	Yes	Increased	N/A	Sodium bicarbonate	N/A
5 (10)	M	1 day	Icterus, weak reactions, hypoglycemia, pneumonia, seizures, and vomiting	Yes	Yes	4092.79 (0–7.6) mmol/mol Cr	Bilateral, symmetric low-intensity areas of cerebral and cerebellar white matter	L-carnitine; vitamins B1, B2, B12, C, and E; and sodium bicarbonate	Severe psychomotor retardation
6 (10)	F	1 year	Dyspnea and vomiting	Yes	Yes	1240.37 (0–7.6) mmol/mol Cr	Normal	L-carnitine, vitamins B1, B12, C, and E	Normal psychomotor development
7 (10)	M	11 days	Dyspnea, poor feeding, diarrhea, and seizures	Yes	Yes	1576.86 (0–7.6) mmol/mol Cr	Left ependymal hemorrhage	L-carnitine; vitamins B1, B2, B12, C, and E; and sodium bicarbonate	Moderate developmental retardation
8 (5)	M	2 days	Jaundice, tachypnea, hyperbilirubinemia, and seizures	Yes	Yes	8723.54 (0–7.6) mmol/mol Cr	N/A	Blood exchange, red blood cell transfusions, sodium bicarbonate, and antioxidants (vitamins C and E)	Died after 18 days
9 (11)	F	1 day	Progressive dyspnea	Yes	Yes	588.941 (reference value 14.922)	Multiple patches of abnormal signals in the subcortex of the frontal lobe (bilateral orientation)	Positive airway pressure ventilation therapy, anti-infection therapy, and alkaline supplementation therapy	Died after 2 months
10 [This study]	F	4 days	Feeding difficulty and tachypnea	Yes	Yes	1100 (normal range: 0–10.0)	A wide range of long T1 and T2 signals in the bilateral frontotemporal parietal lobes, short T1 signals in the bilateral pallidum	L-carnitine; vitamins B1, B2, B12, C, and E; and sodium bicarbonate	Moderate developmental retardation

N/A, data not available; CT, computed tomography; F, female; M, male; MRI, magnetic resonance imaging; Cr, creatinine.

The relationships between genotypes and phenotypes in GSSD remain unclear. However, to some extent, it is possible to predict the clinical severity of the condition based on genetic analysis. Njålsson et al. (16) studied 41 patients with GSSDs from 33 families and found that variants causing frameshift, premature-termination, or aberrant splicing were only observed in patients with moderate or severe clinical phenotypes. In our study, the splicing variant, c.491G > A, accounted for 60.0% (12/20) of the alleles in the 10 reported cases in China, 3 of which were homozygotes and 7 were heterozygotes (Table 1). These nine Chinese patients with the c.491 G > A variant showed moderate to severe clinical phenotypes. Therefore, the c.491 G > A variant in *GSS* is likely the hot spot mutation in the cases observed in China and may be associated with more severe GSSD phenotypes. Notably, the *GSS* c.491G > A variant was first considered to be a missense variant (p.R164Q), but subsequent findings showed that this variant can affect *GSS* mRNA splicing (11–13). More specifically, Shi et al. (13) performed reverse transcriptase-polymerase chain reaction-based amplification combined with Sanger sequencing to confirm that this site variant can lead to exon 5 jumping, resulting in a frameshift as well as premature codon termination. Further, Njålsson et al. (16) showed that in cultured fibroblasts, GSS activity was significantly correlated with GSH levels; however, the difference in GSS activity was insignificant for patients with mild, moderate, or severe GSSD. In recent years, owing to advances in molecular genetic testing, which can be used to quickly and effectively diagnose GSSD, there have been almost no reports of cases where GSSD was diagnosed based on GSH enzyme activity. Therefore, limited data are available for exploring the relationship between enzyme activity and phenotype.

Patients with GSSD require long-term treatment. Unfortunately, however, there are no clinical practice guidelines in this regard. The main treatment strategies include acidosis correction, oxidative stress injury prevention, and the treatment of manifestations. The efficacy of sodium hydrogen carbonate administration to correct acidosis has been confirmed; hence, maintenance therapy with enteral sodium hydrogen carbonate is administered orally after the patient has stabilized (17). Ristoff et al. (2) conducted long-term follow-up of 28 patients with GSSD, and noted that the early administration of large amounts of vitamins C and E may reduce oxidative stress damage and prevent the development of central nervous system symptoms. In addition, it has been observed that selenium prevents oxidative stress by forming selenoproteins; the first patient with a severe phenotype who was treated with selenium showed a good prognosis (18). N-acetylcysteine administration can increase GSH levels in patients with GSSD (19); however, excessive amounts of cysteine are neurotoxic (20). Therefore, N-acetylcysteine is not recommended for patients with high cysteine levels. Tokatli et al. (21) reported a case of acetaminophen-induced hepatotoxicity after 2 days of treatment with regular doses of acetaminophen. Therefore, acetaminophen is also not recommended for patients with GSSD. Moreover, drugs that may induce hemolytic crisis, such as phenobarbital, sulfonamides, and aspirin, should be avoided for patients with GSSD.

The survival prognosis of patients with GSSD largely depends on early diagnosis and treatment (18). Simon et al. (22) suggested the routine inclusion of GSSD in newborn screening panels to identify newborns with milder phenotypes via urine-organic acid analysis or the analysis of dried blood spots using tandem mass spectrometry; this may reduce mortality. Differences in the clinical phenotypes of patients may be influenced by genetic modifiers or environmental factors. In this regard, Njålsson et al. (16) reported that early treatment may delay the progression of moderate GSSD to the severe phenotype. In our case, even though the infant was diagnosed with severe GSSD, her condition improved significantly, and she was generally well at follow-up owing to timely diagnosis and treatment with sodium hydrogen carbonate and antioxidants. When patients present with unexplained hemolytic anemia and metabolic acidosis, the possibility of GSSD should be considered. Furthermore, genetic testing combined with urine-organic acid analysis should be applied promptly to confirm the diagnosis so that treatment can commence as soon as possible, to improve the patient's survival prognosis.

## 4. Conclusions

Here, we report a case of a neonate with severe GSSD carrying compound heterozygous variants in *GSS*, expanding the varietal spectrum. Moreover, we summarize the clinical manifestations, treatment, prognosis, and genetic characteristics of patients with GSSD reported in China to date. Overall, our results indicate that genetic testing may facilitate early GSSD diagnosis and antioxidant therapy may improve prognosis.

## Data Availability

The datasets presented in this study can be found in the Genome Sequence Archive (23) in National Genomics Data Center (24), China National Center for Bioinformation/Beijing Institute of Genomics, Chinese Academy of Sciences (GSA-Human: HRA004967) that are publicly accessible at https://ngdc.cncb.ac.cn/gsa-human
